# CD209 gene polymorphism and its clinical correlation with susceptibility to rheumatoid arthritis among Egyptian patients: A case-control study

**DOI:** 10.1515/rir-2025-0027

**Published:** 2025-12-27

**Authors:** Rasha Mokhtar Elnagar, Amira M. Sultan, Mohammed Elshaer, Alaa A. Awad, Dalia Kamal Nassar

**Affiliations:** Department of Medical Microbiology and Immunology, Faculty of Medicine, Mansoura University, Mansoura 35516, Egypt; Department of Basic Medical Sciences, College of Medicine, AlMaarefa University, Diriyah, 13713, Riyadh, Saudi Arabia; Department of Clinical Pathology, Faculty of Medicine, Mansoura University, Mansoura, Egypt; Department of Physical medicine, Rheumatology and Rehabilitation, Faculty of medicine, Mansoura University, Mansoura, Egypt; Department of Basic Medical Sciences, Faculty of Medicine, Ibn Sina University for Medical Sciences, Amman, Jordan

**Keywords:** rheumatoid arthritis, CD209 polymorphism, Egypt, rs4804803, rs735239

## Abstract

**Background and Objectives:**

Certain genetic traits increase the likelihood of developing rheumatoid arthritis (RA); nevertheless, the association between RA and polymorphisms in the CD209 gene is ambiguous. This study sought to investigate the correlation between RA susceptibility and the CD209 single nucleotide polymorphisms rs4804803 (AG) and rs735239 (AG) within the Egyptian population.

**Methods:**

This case-control study was conducted between January and October 2024 and included 108 participants. Of these, 54 patients were diagnosed with RA according to the 2010 classification criteria of the American College of Rheumatology (ACR) and the European League Against Rheumatism (EULAR). Two CD209 promoter regions rs4804803 (-336A/G) and rs735239 (-871A/G) were genotyped for single nucleotide polymorphisms (SNPs) using multiplex polymerase chain reaction (PCR) and double amplification refractory system (dARMS).

**Results:**

Patients with RA showed significantly higher frequencies of the CD209 rs4804803 SNP in the AG (*P* = 0.036) and GG (*P* = 0.006) genotypes compared to controls. Under the dominant paradigm, those with CD209 rs4804803 genotypes (AG+GG) had an elevated risk of RA (*P* = 0.003). In recessive inheritance model, RA patients had a greater frequency of the rs4804803 GG polymorphism than controls (*P* = 0.017). Additionally, relative to the A allele, the rs4804803 G allele raised the risk of RA (*P* = 0.001). No significant difference was observed between the two groups in the distribution of rs735239 genotypes. However, RA patients carrying the rs4804803 AG or GG genotypes exhibited significantly higher rates of morning stiffness (*P* = 0.001) and elevated CRP levels (*P* = 0.009).

**Conclusions:**

This work highlights the significant role of the CD209 rs4804803 polymorphism, particularly the G allele, in the elevated susceptibility to RA among Egyptians.

## Introduction

Rheumatoid arthritis (RA) is a chronic autoimmune disorder that mainly affects diarthrodial joints.^[1]^ It is characterized by complex interactions among immune cells, and inflammatory mediators inside the joint milieu.^[2]^ Amongst these immune cells are macrophages and dendritic cells (DCs) that proliferate and infiltrate the synovium, where they activate T and B lymphocytes cells and secrete proinflammatory cytokines.^[3]^ These processes contribute significantly to the pathophysiology of RA, resulting in cartilage degradation, bone deterioration, discomfort, and joint stiffness.^[4]^ Genetics also play an important role, with polymorphisms in the major histocompatibility complex (MHC) accounting for over 60% of RA susceptibility. In addition, almost 100 non-MHC genes have been linked to RA development.^[5]^ One gene of relevance is CD209, also known as dendritic cell-specific intercellular adhesion molecule-3-grabbing non-integrin (DC-SIGN). CD209 is a type II C-type lectin receptor (CLR), a class of pattern recognition receptors located on DCs and macrophages, involved in cell adhesion and immune signaling.^[6,7]^ DC-SIGN participates in T cell activation, antigen acquisition, and dendritic cell migration. However, certain pathogens and tumor cells can exploit DC-SIGN to evade immune recognition, resulting in immune suppression.^[8,9]^

In the context of RA, CD209 is highly expressed on synovial macrophages (CD68^⁺^) and functions as a key signaling molecule involved in regulating monocyte-driven T cell activation. Increased CD209 expression on macrophages and DCs correlates with the degree of cartilage and bone degradation in RA patients, a correlation not found in persons with osteoarthritis or traumatic joint injuries.^[10,11]^ Although the precise mechanisms remain unclear, single nucleotide polymorphisms (SNPs) in the promoter region of the CD209 gene are thought to modulate its expression, potentially altering immune responses and thereby increasing susceptibility to chronic inflammatory conditions, including RA, colitis, Crohn‘s disease, and Kawasaki disease. Additionally, CD209 SNPs are associated with increased vulnerability to several infectious diseases, including human immunodeficiency virus type 1 (HIV-1),^[12]^ and hepatitis C virus (HCV),^[13]^ which have also been suggested to accelerate autoimmune processes such as RA development.^[14]^ Moreover, CD209 polymorphisms have been linked to type 1 diabetes mellitus^[15]^ and pulmonary tuberculosis.^[16]^

Despite the worldwide interest in CD209 gene variants and their potential contribution in autoimmune pathogenesis, their function in RA susceptibility in North African populaces, particularly Egyptians, remains largely unexplored and information relevant to Egyptian RA patients is scarce. Given the immunological significance of CD209 and the distinct genetic profile of autoimmune diseases in the Egyptian population, this study investigated the relationship between CD209 gene polymorphisms and susceptibility to RA in Egyptian patients, alongside assessing how these genetic variations correlate with disease severity and progression.

## Materials and Methods

### Study Subjects and Design

This ten-month prospective case-control study was conducted from January to October 2024. Atotal of 108 participants were included: 54 RA patients and 54 healthy controls. The RA group consisted of patientswho fulfilled the 2010 classification criteria for RA established by the American College of Rheumatology (ACR) /European League Against Rheumatism (EULAR). They were consecutively recruited from the outpatient clinics of the Physical Medicine, Rheumatology, and Rehabilitation department at Mansoura University Hospital, Egypt. The control group consisted of 54 healthy participants with no previous history of autoimmune, inflammatory, or chronic systemic diseases. Controls were chosen from hospital staff and blood donors following medical screening to ensure eligibility. Although individual matching was not executed, controls were selected to closely match the RA group in terms of age and gender distribution. Exclusion criteria for both groups included the presence of autoimmune diseases other than RA, malignancy, diabetes mellitus, impaired renal or hepatic function, pregnancy or lactation, chronic infections, cardiovascular disease, and endocrine disorders. All RA patients underwent thorough history-taking and clinical examination, including assessment of demographics, clinical signs and symptoms, affected joints, current anti-RA medications, functional impairment, disease activity score (DAS28), severity index, history of joint surgery, and abnormal radiologic findings. Nervous system involvement was characterized by the presence of peripheral neuropathy, diagnosed through patient-reported neurological symptoms (sensory and/or motor) and comprehensive neurological examinations in all patients, with nerve conduction studies conducted in select cases for confirmation when necessary. Laboratory evaluation included Rheumatoid Factor (RF), Erythrocyte Sedimentation Rate (ESR), C-reactive protein (CRP), platelet count, and anti-cyclic citrullinated peptide (anti-CCP) antibodies.

### Samples Collection and Genomic DNA Extraction

Three milliliters of whole blood were collected from each participant into EDTA tubes. Genomic DNA was extracted from peripheral blood leukocytes using the Gene-Fast DNA Extraction Kit (ViroGene, Egypt) at the Immunology and Genetic unit, Department of Medical Microbiology and Immunology, Faculty of Medicine, Mansoura University. Extracted DNA was stored at-20°C for subsequent analysis of CD209 promoter SNPs, following the manufacturer‘s instructions.

### CD209 (Rs4804803/rs735239) SNPs Molecular Analysis

Two CD209 promoter regions rs4804803 (-336AG) and rs735239 (-871AG) were genotyped for SNPs using multiplex polymerase chain reaction (PCR) combined with the double amplification refractory system (dARMS), as previously described.^[17,18]^ The following primer sequences were used: rs4804803-Forward: 5′- CACATCATCTCATCTGGAC-3′, rs4804803-Reverse: 5′-GATTGGAATACTATACAGC-3′, and rs735239- Forward: 5′- GTTAGCTAAACTTGCAGTGC-3′, rs735239-Reverse: 5′- CCAC AGCTTTTATTTCCCAC-3′). The PCR reactions were carried out at a final volume of 25 μL, including 5 μL of genomic DNA, 1 μL of each primer (Sigma-Aldrich, UK), 12.5 μL of Taq polymerase (COSMO PCR Master Mix, Willowfort. co., UK), and 3.5 μL of nuclease free water. The thermocycling settings were as follows: initial denaturation at 95°C for 3 min, followed by thirty-five amplification cycles consisting of (denaturation at 95°C for 45 seconds, annealing at 60°C for 1 min, and extension at 72°C for 1 min), and a final extension at 72°C for 7 min. The PCR-amplified DNA fragements were resolved on a 2.5% agarose gel and visualized under UV illumination.

### Statistical Analysis

Data were analyzed using SPSS version 25.0 (IBM Corp., Armonk, NY, USA). Relationships between categorical variables were evaluated with the Chi-square test, applying the Monte Carlo method when the expected cell counts were low. Differences in group means were assessed using the Student‘s *t*-test for two groups, and one-way ANOVA for comparisons among three or more groups. For non-parametric data, the Kruskal-Wallis test was performed to determine statistical significance among more than two groups. The Hardy–Weinberg equilibrium was assessed in each group to confirm population genetic stability. Haplotypes were estimated using HaploView version 4.2, which applies the expectation maximization (EM) algorithm, and the goodness-of-fit between observed and expected genotype frequencies was evaluated.^[19]^ Logistic and ordinal regression analyses were carried out to identify potential risk factors. Statistical significance was set at a *p*-value ≤ 0.05, with a 95% confidence interval.

## Results

### Characteristics of the Study Participants

This study enrolled 108 participants, divided equally into two groups: 54 patients with RA and 54 healthy controls. The age and sex distributions did not differ considerably between the groups. Among RA patients, the mean age at disease onset was 32.6 years (range: 1–57), and the average disease duration was 13.6 years (range: 1–35). Morning stiffness (MS) lasting less than 60 min was seen in most RA patients (81.5%, *n* = 44), with a median duration of 17.5 min (range: 10–60). Neuropathy was detected in 23 out of 54 RA patients (42.6%). After further assessment, 18 of these cases (78.3%) were deemed RA-related, based on clinical findings and laboratory results indicative of immune-mediated or vasculitic neuropathy. Five cases (21.7%) were ascribed to non-RA-related causes, predominantly cervical disc disease (*n* = 3) and other unrelated conditions (*n* = 2). Arthritis involving three or more joint regions was observed in 51.9% of patients (*n* = 28), while all patients (100%) had arthritis of the hand joints. Additional clinical manifestations were rheumatoid nodules (7.4%, *n* = 4) and cutaneous vasculitis (3.7%, *n* = 2). The Disease Activity Score 28-CRP (DAS28-CRP) was evaluated for all participants, yielding a median of 3.3, with a range from 2.3 to 7.5. Only 5.6% (*n* = 3) attained remission, 42.6% (*n* = 23) exhibited low activity, 20.4% (*n* = 11) shown moderate activity, and 31.5% (*n* = 17) presented high activity. The median Visual Analogue Scale (VAS) was 5, with a range from 2 to 8. Both RF and CRP were positive in 90.7% (*n* = 49) of RA patients, with CRP values exhibiting a median of 17 and a range of 6 to 96 mg/L. The median ESR was 36.5 mm/hr, with a range of 6 to 11 mm/hr. Anti-CCP antibodies were detected in 90.7% (*n* = 49) of patients. Non-steroidal anti-inflammatory medicines (NSAIDs), conventional disease-modifying antirheumatic drugs (cDMARDs), and corticosteroids were given to 98.1% (*n* = 53) of the RA patients, whereas 42.6% (*n* = 23) received biological treatment (Table 1).

**Table 1:** Demographic, clinical and laboratory aspects of the study participants

Variables	RA (*n* = 54)	Control (*n* = 54)	*P*-value
Gender			
Male	12 (22.2%)	18 (33.3%)	χ^2^, 0.197
Female	42 (77.8%)	36 (66.7%)	
Age (years)			
Mean ± SD.	46.2 ± 11.1	49.8 ± 16.2	*t*, 0.175
Median (Min. – Max. )	46.5 (25 – 67)	51 (14 – 88)	
Age of onset (years)			
Mean ± SD.	32.6 ± 12.7	—	—
Median (Min. – Max. )	32 (1 – 57)	—	
Duration (years)			
Mean ± SD.	13.6 ± 9.21	—	—
Median (Min. – Max. )	12 (1 – 35)	—	
Symptoms			
Morning stiffness (min)			
>60	10 (18.5%)	—	—
≤60	44 (81.5%)	—	
Mean ± SD.	25.2 ± 15.3	—	
Median (Min. – Max. )	17.5 (10 – 60)	—	
Rheumatoid nodules	4 (7.4%)	—	
Cutaneous vasculitis	2 (3.7%)	—	
Neuropathy	23 (42.6%)	—	
RA-related	18 (78.3%)	—	
Non-RA- related	5 (21.7%)	—	
Arthritis of 3 or more joint areas	28 (51.9%)	—	
Arthritis of hand joints	54 (100%)	—	
DAS28-CRP			
<2.6 disease remission	3 (5.6%)	—	—
2.6- 3.2 Low activity	23 (42.6%)	—	
>3.2- 5.1 Moderate activity	11 (20.4%)	—	
>5.1 high activity	17 (31.5%)	—	
Mean ± SD.	4.13 ± 1.59	—	
Median (Min. – Max. )	3.27 (2.3 – 7.51)	—	
VAS scale			
Mean ± SD.	4.98 ± 2	—	—
Median (Min. – Max. )	5 (2 – 8)	—	
ESR (mm/h)			
Mean ± SD.	43.4 ± 24.8	—	—
Median (Min. – Max. )	36.5 (6 – 110)	—	
RF	49 (90.7%)	—	
Positive CRP (mg/L)	49 (90.7%)	—	
Mean ± SD.	28.7 ± 27.4	—	
Median (Min. – Max. )	17 (6 – 96)	—	
Anti-CCP	49 (90.7%)	—	
Treatment			
NSAIDS	53 (98.1%)	—	—
cDMARDs	53 (98.1%)	—	
Steroids	53 (98.1%)	—	
Biological therapy	23 (42.6%)	—	

SD, standard deviation; Min, minimum; Max, maximum; χ^2^, chi square test; *t*, student *t* test; *, statistically significant; Categorical data was expressed by using count (%). —, no data. RA-related and Non-RA-related percentages are calculated relative to the total neuropathy cases (*n* = 23) within RA patients.

### Genotypes and Haplotypes of CD209 Promoter Region SNPs

**Figure 1:**
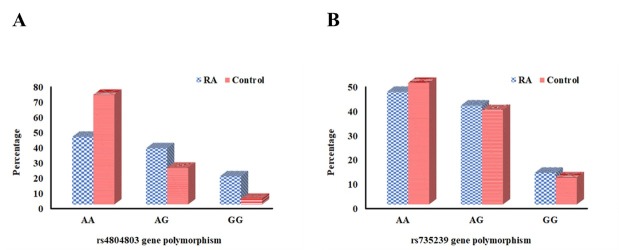
Distribution of CD209 polymorphism in RA patients versus controls. (A) rs4804803 genotype. (B) rs735239 genotype.

The SNPs in the CD209 promoter region were examined in both study groups. In RA patients, the genotypes AG (OR = 1.77, 95% CI: 1.04–3.03, *P* = 0.036) and GG (OR = 3.56, 95% CI: 1.45–8.77, *P* = 0.006), along with the G allele (OR = 2.04, 95% CI: 1.37–3.02, *P* < 0.001) of the rs4804803 SNP, were significantly more prevalent than in controls, suggesting a potential correlation with heightened RA risk. No marked dissimilarities in genotype or allele occurrences for the rs735239 SNP were identified between the two groups. Haplotype analysis of CD209 rs4804803 and rs735239 demonstrated significant correlations with RA for the AG haplotype (OR = 5.07, 95% CI: 1.73–14.90, *P* = 0.003) and the GG haplotype (OR = 4.21, 95% CI: 1.33–13.31, *P =* 0.014), indicating a synergistic impact of these variations on disease susceptibility. These findings highlight the possible role of CD209 genetic variants in the etiology of RA. The distribution of CD209 SNP genotypes, alleles, and haplotypes in the study groups is depicted in Table 2 and Figure 1.

**Table 2:** CD209 genotypes, alleles and haplotypes distribution among RA patients and control groups

			RA (*n* = 54)	Control (*n* = 54)	*P*	OR (95% CI)
rs4804803	Genotypes	AA	24 (44.4%)	39 (72.2%)		Reference
AG polymorphism		AG	20 (37.0%)	13 (24.1%)	0.036^*^	1.77 (1.04–3.03)
		GG	10 (18.5%)	2 (3.7%)	0.006^*^	3.56 (1.45–8.77)
	Dominant model	AA	24 (44.4%)	39 (72.2%)		Reference
		AG+GG	30 (55.6%)	15 (27.8%)	0.003^*^	2.08 (1.27–3.41)
	Recessive model	AA+AG	44 (81.5%)	52 (96.3%)		Reference
		GG	10 (18.5%)	2 (3.7%)	0.017^*^	2.92 (1.21–7.05)
	Allele	A	68 (63.0%)	91 (84.3%)		Reference
		G	40 (37.0%)	17 (15.7%)	<0.001^*^	2.04 (1.37–3.02)
		HWE	0.130	0.497		
rs735239	Genotypes	AA	25 (46.3%)	27 (50.0%)		Reference
AG polymorphism		AG	22 (40.7%)	21 (38.9%)	0.765	1.08 (0.65–1.79)
		GG	7 (13.0%)	6 (11.1%)	0.710	1.16 (0.54–2.48)
	Dominant model	AA	25 (46.3%)	27 (50.0%)		Reference
		AG+GG	29 (53.7%)	27 (50.0%)	0.700	1.10 (0.68–1.76)
	Recessive model	AA+AG	47 (87.0%)	48 (88.9%)		Reference
		GG	7 (13.0%)	6 (11.1%)	0.767	1.12 (0.54–2.31)
	Allele	A	72 (66.7%)	75 (69.4%)		Reference
		G	36 (33.3%)	33 (30.6%)	0.662	1.08 (0.76–1.55)
		HWE	0.540	0.539		
rs4804803- rs735239	haplotypes	AA	0.544	0.852		Reference
		AG	0.181	0.023	0.003^*^	5.07 (1.73-14.90)
		GA	0.154	0.102	0.203	1.58 (0.78-3.28)
		GG	0.121	0.023	0.014^*^	4.21 (1.33-13.31)

Reference was defined based on NCBI databases; Genotypes and alleles are expressed by using count (%); haplotypes are expressed using frequency. OR, odds ratio; CI, confidence interval; HWE, Hardy Weinberg equilibrium; *, statistically significant.

**Figure 2:**
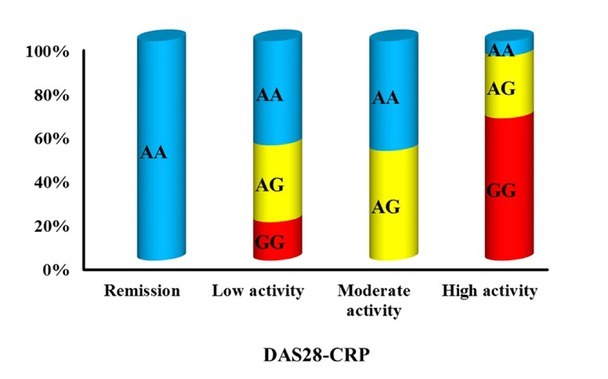
Association between rs4804803 gene polymorphism and DAS28-CRP among RA.

Correlation examination of CD209 polymorphisms with several demographic, clinical, and laboratory factors in the RA patient cohort indicated that the rs4804803 polymorphism, particularly the AG and GG genotypes, was strongly linked to more severe disease characteristics. The findings encompassed a greater prevalence of arthritis impacting over three joint regions (*P* = 0.001), prolonged MS duration (*P* = 0.001), higher DAS28-CRP scores (*P* = 0.003), increased VAS scores (*P* < 0.001), elevated CRP levels (*P* = 0.009), raised ESR (*P* = 0.012), and an augmented requirement for biologic therapy (*P* = 0.001) (Table 3, Figure 2). The rs735239 polymorphism exhibited no significant correlation with any of the evaluated demographic, clinical, or laboratory data, as outlined in Table 4.

**Table 3:** Association between genotypes and different parameters among RA group with rs4804803 gene polymorphism

	rs4804803 AG gene polymorphism			*P*-value
	AA (*n* = 24)	AG (*n* = 20)	GG (*n* = 10)	
Gender				
Male	4 (16.7%)	5 (25%)	3 (30%)	χ^2^, 0.698
Female	20 (83.3%)	15 (75%)	7 (70%)	
Age (years)				
Mean ± SD.	48 ± 9.88	43.3 ± 11.3	47.5 ± 13.3	*F*, 0.345
Median (Min. – Max. )	47.5 (32 – 65)	42 (25 – 67)	48.5 (25 – 65)	
Age of onset (years)				
Mean ± SD.	37.2 ± 10.7	28.7 ± 8.8	29.5 ± 20	*F*, 0.058
Median (Min. – Max. )	35.5 (19 – 56)	27 (16 – 50)	24.5 (1 – 57)	
Duration (years)				
Mean ± SD.	10.8 ± 8.49	14.6 ± 8.48	18 ± 10.9	H, 0.110
Median (Min. – Max. )	7 (1 – 30)	13.5 (1 – 32)	18 (1 – 35)	
Morning stiffness (min)				
>60	1 (4.2%)	3 (15%)	6 (60%)	χ^2^, 0.001^*^
≤60	23 (95.8%)	17 (85%)	4 (40%)	
Mean ± SD.	22.6 ± 14.8	26.2 ± 14.1	36.3 ± 21.4	H, 0.341
Median (Min. – Max. )	15 (10 – 60)	30 (10 – 60)	37.5 (10 – 60)	
Rheumatoid nodules	1 (4.2%)	2 (10%)	1 (10%)	χ^2^, 0.657
Cutaneous vasculitis	1 (4.2%)	1 (5%)	0 (0%)	χ^2^, 1.0
Neuropathy	12 (50%)	7 (35%)	4 (40%)	χ^2^, 0.595
RA-related	9 (75%)	6 (85.7%)	3 (75%)	
Non-RA-related	3 (25%)	1 (14.3%)	1 (25%)	
Arthritis of 3 or more joint areas	6 (25%)	13 (65%)	9 (90%)	χ^2^, 0.001^*^
DAS28-CRP				
<2.6 disease remission	3 (12.5%)	0 (0%)	0 (0%)	χ^2^, 0.003^*^
2.6- 3.2 Low	13 (54.2%)	8 (40%)	2 (20%)	
>3.2- 5.1 Moderate	6 (25%)	5 (25%)	0 (0%)	
>5.1 High	2 (8.3%)	7 (35%)	8 (80%)	
Mean ± SD.	3.22 ± 0.89	4.33 ± 1.44	5.94 ± 1.58	H, <0.001^*^
Median (Min. – Max. )	2.81 (2.3 – 5.37)	3.69 (2.9 – 7.08)	6.45 (3.2 – 7.51)	
VAS scale				
Mean ± SD.	3.63 ± 1.35	5.55 ± 1.88	7.1 ± 0.99	H <0.001^*^
Median (Min. – Max. )	3.5 (2 – 7)	6 (2 – 8)	7 (5 – 8)	
RF	22 (91.7%)	18 (90%)	9 (90%)	χ^2^, 1.0
Positive CRP (mg/L)	20 (83.3%)	19 (95%)	10 (100%)	χ^2^, 0.316
Mean ± SD.	14.5 ± 10.6	34.8 ± 28.1	45.3 ± 36.8	H, 0.009^*^
Median (Min. – Max. )	12 (6 – 50)	24 (6 – 96)	28.5 (6 – 96)	
ESR (mm/h)				
Mean ± SD.	32.9 ± 15	44.9 ± 25.1	65.4 ± 29.6	H, 0.012^*^
Median (Min. – Max. )	34.5 (10 – 75)	36 (6 – 98)	71 (22 – 110)	
Anti-CCP	22 (91.7%)	18 (90%)	9 (90%)	χ^2^, 1.0
Treatment				
NSAIDS	24 (100%)	20 (100%)	9 (90%)	χ^2^, 0.191
Biological therapy	4 (16.7%)	11 (55%)	8 (80%)	χ^2^, 0.001*
cDMARDs	24 (100%)	20 (100%)	9 (90%)	χ^2^, 0.191
Steroids	24 (100%)	20 (100%)	9 (90%)	χ^2^, 0.191

χ^2^, chi square test; MC, Monte Carlo; *F*, One Way ANOVA test; H, Kruskal Wallis test; SD, standard deviation; *, statistically significant. RA-related and Non-RA-related percentages are calculated relative to the total neuropathy cases within each genotype.

**Table 4:** Relationship between genotypes and different parameters among RA group with rs735239 gene polymorphism

	rs735239 AG gene polymorphism			*P*-value
	AA (*n* = 25)	AG (*n* = 22)	GG (*n* = 7)	
Gender				
Male	7 (28%)	3 (13.6%)	2 (28.6%)	χ^2^, 0.536
Female	18 (72%)	19 (86.4%)	5 (71.4%)	
Age (years)				
Mean ± SD.	47 ± 10.6	44.3 ± 10.1	49.4 ± 15.9	*F*, 0.512
Median (Min. – Max. )	44 (27 – 67)	44.5 (25 – 65)	53 (25 – 65)	
Age of onset (years)				
Mean ± SD.	34.8 ± 10.5	29.4 ± 14.7	35 ± 13.2	*F*, 0.310
Median (Min. – Max. )	34 (20 – 57)	26.5 (1 – 56)	30 (19 – 56)	
Duration (years)				
Mean ± SD.	12.2 ± 9.28	14.9 ± 9.24	14.3 ± 9.55	H, 0.535
Median (Min. – Max. )	10 (1 – 32)	13.5 (2 – 35)	18 (2 – 23)	
Morning stiffness (min)				
>60	2 (8%)	6 (27.3%)	2 (28.6%)	χ^2^, 0.148
≤60	23 (92%)	16 (72.7%)	5 (71.4%)	
Mean ± SD.	26.7 ± 13.7	22.8 ± 17.4	26 ± 17.5	H, 0.292
Median (Min. – Max. )	30 (10 – 60)	15 (10 – 60)	15 (10 – 45)	
Rheumatoid nodules	2 (8%)	1 (4.5%)	1 (14.3%)	χ^2^, 0.622
Cutaneous vasculitis	1 (4%)	1 (4.5%)	0 (0%)	χ^2^, 1.0
Neuropathy	12 (48%)	10 (45.5%)	1 (14.3%)	χ^2^, 0.311
RA related	9 (75%)	8 (80%)	1 (100%)	
Non- RA-related	3 (25%)	2 (20%)	0 (0%)	
Arthritis of 3 or more joint areas	12 (48%)	13 (59.1%)	3 (42.9%)	χ^2^, 0.696
DAS28-CRP				
<2.6 disease remission	1 (4%)	1 (4.5%)	1 (14.3%)	
2.6- 3.2 Low	12 (48%)	8 (36.4%)	3 (42.9%)	χ^2^, 0.799
>3.2- 5.1 Moderate	6 (24%)	4 (18.2%)	1 (14.3%)	
>5.1 High	6 (24%)	9 (40.9%)	2 (28.6%)	
Mean ± SD.	3.95 ± 1.49	4.44 ± 1.76	3.82 ± 1.4	H, 0.454
Median (Min. – Max. )	3.13 (2.5 – 7.08)	3.49 (2.3 – 7.51)	3.2 (2.4 – 5.71)	
VAS scale				
Mean ± SD.	4.72 ± 2.01	5.27 ± 2.1	5 ± 1.83	H, 0.667
Median (Min. – Max. )	4 (2 – 8)	5.5 (2 – 8)	5 (2 – 7)	
RF	23 (92%)	20 (90.9%)	6 (85.7%)	χ^2^, 0.835
Positive CRP (mg/L)	24 (96%)	20 (90.9%)	5 (71.4%)	χ^2^, 0.150
Mean ± SD.	24.7 ± 22	28.1 ± 28.2	50.4 ± 42.1	H, 0.245
Median (Min. – Max. )	12 (6 – 86)	19 (6 – 96)	30 (12 – 96)	
ESR (mm/h)				
Mean ± SD.	45.7 ± 23.4	40.8 ± 25.7	43 ± 29.6	H, 0.768
Median (Min. – Max. )	38 (20 – 98)	35.5 (6 – 110)	40 (12 – 83)	
Anti-CCP	24 (96%)	19 (86.4%)	6 (85.7%)	χ^2^, 0.335
Treatment				
NSAIDS	25 (100%)	21 (95.5%)	7 (100%)	χ^2^, 0.533
Biological therapy	10 (40%)	10 (45.5%)	3 (42.9%)	χ^2^, 0.929
cDMARDs	25 (100%)	21 (95.5%)	7 (100%)	χ^2^, 0.533
Steroids	25 (100%)	21 (95.5%)	7 (100%)	χ^2^, 0.533

χ^2^, chi square test; MC, Monte Carlo; *F*, One Way ANOVA test; H, Kruskal Wallis test; SD, standard deviation; *, statistically significant. RA-related and Non-RA-related percentages are calculated relative to the total neuropathy cases within each genotype.

### Association of DAS28-CRP with Clinical and Laboratory Features in RA

A comparison of DAS28-CRP scores with clinical and laboratory criteria among RA patients revealed that elevated disease activity grades were significantly correlated with arthritis affecting three or more joint regions, extended MS, increased VAS scores, elevated CRP, high ESR, and the administration of biologic therapy (*P*-values < 0.001), as well positive anti-CCP antibodies (*P* = 0.005) (Table 5).

**Table 5:** Association between DAS28-CRP and different parameters among RA group

	DAS28-CRP				*P*-value
	Remission (*n* = 3)	Low (*n* = 23)	Moderate (*n* = 11)	High (*n* = 17)	
Gender					
Male	0 (0.0%)	5 (21.7%)	2 (18.2%)	5 (29.4%)	χ^2^, 0.884
Female	3 (100.0%)	18 (78.3%)	9 (81.8%)	12 (70.6%)	
Age (years)					
Mean ± SD.	49.7 ± 14.6	47.6 ± 9.66	40.6 ± 10.3	47.3 ± 12.6	
Median (Min. – Max. )	48 (36 – 65)	47 (32 – 67)	41 (25 – 58)	51 (25 – 65)	*F*, 0.317
Age of onset (years)					
Mean ± SD.	33 ± 8.54	33.4 ± 11.7	29.5 ± 10.6	33.5 ± 16.1	*F*, 0.842
Median (Min. – Max. )	32 (25 – 42)	33 (4 – 56)	26 (16 – 51)	32 (1 – 57)	
Duration (years)					
Mean ± SD.	16.7 ± 11	14.1 ± 9.46	11.2 ± 9.15	13.8 ± 9.19	H, 0.838
Median (Min. – Max. )	23 (4 – 23)	15 (1 – 32)	7 (1 – 30)	13 (1 – 35)	
Morning stiffness (min)					
>60	0 (0%)	0 (0%)	1 (9.1%)	9 (52.9%)	χ^2^, <0.001*
≤60	3 (100%)	23 (100%)	10 (90.9%)	8 (47.1%)	
Mean ± SD.	13.3 ± 2.89	21.7 ± 12.9	23.5 ± 11.1	41.9 ± 18.3	H, 0.015*
Median (Min. – Max. )	15 (10 – 15)	15 (10 – 60)	22.5 (10 – 45)	45 (15 – 60)	
Rheumatoid nodules	0 (0%)	0 (0%)	0 (0%)	4 (23.5%)	χ^2^, 0.031*
Cutaneous vasculitis	0 (0%)	0 (0%)	1 (9.1%)	1 (5.9%)	χ^2^, 0.368
Neuropathy (RA-related)	2 (66.7%)	6 (26.1%)	5 (45.5%)	5 (29.4%)	χ^2^, 0.410
Arthritis of 3 or more joint areas	0 (0%)	6 (26.1%)	5 (45.5%)	17 (100%)	χ^2^, <0.001*
VAS scale					
Mean ± SD.	3.33 ± 0.58	3.7 ± 1.36	4.64 ± 1.69	7.24 ± 0.75	H, <0.001*
Median (Min. – Max. )	3 (3 – 4)	4 (2 – 7)	4 (2 – 7)	7 (6 – 8)	
RF	1 (33.3%)	22 (95.7%)	9 (81.8%)	17 (100%)	χ^2^, 0.005*
Positive CRP (mg/L)	2 (66.7%)	20 (87%)	10 (90.9%)	17 (100%)	χ^2^, 0.169
Mean ± SD.	9 ± 4.24	11.6 ± 5.16	27.4 ± 19.2	51.9 ± 32	
Median (Min. – Max. )	9 (6 – 12)	12 (6 – 21)	26 (6 – 60)	48 (12 – 96)	H, <0.001*
ESR (mm/h)					
Mean ± SD.	19.7 ± 10.8	30.4 ± 12.7	42.3 ± 20.6	65.8 ± 25.5	H, <0.001*
Median (Min. – Max. )	15 (12 – 32)	32 (6 – 63)	37 (23 – 89)	70 (25 – 110)	
Anti-CCP	1 (33.3%)	22 (95.7%)	9 (81.8%)	17 (100%)	χ^2^, 0.005*
Treatment					
NSAIDS	3 (100%)	22 (95.7%)	11 (100%)	17 (100%)	χ^2^, 1.0
Biological therapy	0 (0%)	2 (8.7%)	4 (36.4%)	17 (100%)	χ^2^, <0.001*
cDMARDs	3 (100%)	22 (95.7%)	11 (100%)	17 (100%)	χ^2^, 1.0
Steroids	3 (100%)	22 (95.7%)	11 (100%)	17 (100%)	χ^2^, 1.0

χ^2^, chi square test; MC, Monte Carlo; *F*, One Way ANOVA test; H, Kruskal Wallis test; *, statistically significant. RA-related neuropathy percentages include only neuropathy cases attributed to RA after clinical and laboratory assessment. Non-RA-related cases (*n* = 5) are excluded. Percentages are calculated relative to the total number of patients within each DAS28-CRP category.

Regression analysis models were used to determine determinants of elevated DAS28-CRP values in RA patients with

the rs4 804 803 AG polymorphism. In univariable analysis, significant correlations were identified with prolonged MS duration (OR = 9.60, 95% CI: 3.08–29.94, *P* < 0.001), increased RF (OR = 3.07, 95% CI: 1.11–8.52, *P* = 0.031), and the existence of positive anti-CCP antibodies (OR = 3.07, 95% CI: 1.11–8.52, *P* = 0.031). However, in multivariate analysis, only extended MS duration was identified as an independent predictor of elevated DAS28-CRP scores in patients with AG and GG genotypes of rs4804803 (OR = 6.24, 95% CI: 1.94–20.10, *P* = 0.002)(Table 6).

**Table 6:** Regression analysis for prediction of DAS28-CRP among RA patients with rs4804803 AG and GG genotypes (n = 30)

	Univariate		Multivariate	
	*P*-value	OR (95% CI)	*P*-value	OR (95% CI)
Age	0.640	0.99 (0.97–1.02)		
Gender	0.326	1.43 (0.70–2.94)		
Age of onset	0.953	0.999 (0.98–1.02)		
Duration	0.631	0.99 (0.96–1.02)		
Morning stiffness	<0.001*	9.60 (3.08–29.94)	0.002*	6.24 (1.94–20.10)
RF	0.031*	3.07 (1.11–8.52)	0.458	1.88 (0.35–10.01)
Positive CRP	0.051	2.80 (0.99–7.88)		
Anti-CCP	0.031*	3.07 (1.11–8.52)	0.458	1.88 (0.35–10.01)

OR, odds ratio; CI, confidence interval; *, statistically significant.

## Discussion

Evidence indicates that CD209 is implicated in autoimmune pathways related to RA, facilitating the differentiation of follicular T helper cells and enhancing interleukin (IL)-27 production, which subsequently exacerbates joint inflammation and injury by activating fibroblast-like synoviocytes (FLSs), key effector cells in RA pathogenesis.^[20]^ Prior research indicates that SNPs in the CD209 gene may influencee the severity of RA,^[21]^ although their correlation with RA susceptibility has not been explored in Egypt. Given the potential role of CD209 promoter variants in immune regulation, this case-control study examined the association between CD209 polymorphisms, rs4804803 (-336G > A) and rs735239 (-871G > A), and RA susceptibility in an Egyptian population, while also assessing their association with clinical disease characteristics. These SNPs were chosen because of their elevated prevalence among Egyptians and their established correlations with other diseases in our community.

Our study demonstrates a significant association between the CD209 rs4804803 polymorphism and RA susceptibility in Egyptians. Specifically, the heterozygous AG and homozygous GG genotypes were strongly correlated with increased RA risk. The GG genotype was present in 18.5% of RA patients compared to 3.7% of healthy controls (*P* = 0.006), while the AG genotype was detected in 37.0% of RA patients versus 24.1% of controls (*P* = 0.036). Inheritance model analysis indicated that the dominant model (AG+GG) and recessive model (GG) were both significantly associated with RA (*P* = 0.003; 0.017, respectively), while the G allele was associated with a two-fold greater risk (*P* = 0.001). No notable differences were observed for the CD209 rs735239 polymorphism. Haplotype analysis of rs4804803 and rs735239 demonstrated significant associations with RA for the AG (*P* = 0.003) and GG (*P* = 0.014) haplotypes, indicating a possible cumulative effect of these variants. These findings support a role for CD209 as a genetic susceptibility factor in RA.

Comparative analyses across populations demonstrated both concordance and divergence regarding the role of CD209 polymorphisms in RA susceptibility. In our cohort, the rs4804803 G allele was significantly associated with an increased risk of RA, a finding that aligns with an earlier Colombian investigation reporting a similar association between the CD209 rs4804803 (–336G > A) promoter variant and higher RA risk.^[22]^ Conversely, a recent Taiwanese study, which examined a broader set of CD209 SNPs, reported that individuals carrying the T/T genotype at rs7359874 and the C/A genotype at rs558555834 exhibited a markedly increased risk of RA, whereas no significant associations were observed for rs735240T, rs735239C, rs4804803C, and rs2287886C.^[17]^ These results highlight the potential role of CD209 in RA pathogenesis but also demonstrate the complexity of genetic contributions, as findings differ markedly across populations. Similarly, Díeguez-González *et al*. found no significant associations between CD209 polymorphisms and RA susceptibility in a Spanish cohort.^[23]^ These heterogeneous findings suggest that the contribution of CD209 polymorphisms to RA susceptibility is influenced by ethnic diversity, environmental exposures, and underlying genetic backgrounds, including differences in allele frequencies and linkage disequilibrium patterns across populations. This variability underscores the importance of population-specific genetic investigations to better understand the interaction between CD209 variants, immune regulation, and RA pathogenesis.

In this study, most RA patients were females (77.8%), 51.9% had polyarticular joint involvement, and all patients exhibited hand joint affection. Subcutaneous nodules were detected in 7.4% of cases, while 90.7% tested positive for RF, CRP, and anti-CCP. The mean age of disease onset was 32.6 ± 12.7 years, with an average disease duration of 13.6 ± 9.21 years. This onset age is considerably younger than that commonly reported in international studies, where RA typically manifests around 55 years or later. However, our findings are consistent with a nationwide Egyptian study involving 26 rheumatology centers, which reported a mean onset age of 38.4 ± 11.6 years.^[24]^ This suggests a tendency toward earlier onset in Egyptian cohorts, potentially influenced by genetic

and environmental predispositions, differences in healthcare access and diagnostic practices, as well as region-specific sociodemographic and occupational risk factors. These clinical and serological findings in our cohort were comparable to those reported in previous investigations conducted in diverse populations.^[21,25]^

In our cohort, the prevalence of neuropathy was higher than that reported in most international studies. This difference may be partly explained by our setting as a tertiary referral hospital, where patients often present with more complex disease, and by the systematic neurological assessments performed in our clinic, which likely enabled better detection compared with centers where such evaluations are not routinely conducted.

Significant correlations were observed between the CD209 rs4804803 polymorphism and several clinical and laboratory parameters. Patients with the AA and AG genotypes displayed a longer duration of morning stiffness (*P* = 0.001), higher CRP and ESR levels (*P* = 0.009 and 0.012, respectively), involvement of more than three joints (*P* = 0.001), and elevated disease activity scores (DAS28-CRP, *P* = 0.003). Moreover, these individuals were more likely to require biologic treatment (*P* = 0.001), suggesting a potential association between the rs4804803 variant and more severe RA phenotypes. In contrast, no significant associations were observed for the CD209 rs735239 genotype with any clinical or laboratory parameters, including biologic treatment requirements. Additionally, no gender-specific differences were detected for either rs4804803 or rs735239 polymorphisms in our cohort. Nevertheless, Cáliz *et al*. reported a gender-based effect, with male carriers of the CD209 rs4804803 G allele having a reduced incidence of RA, which was not observed in female carriers.^[21]^

Despite some of the key references on rs4804803 being relatively dated, there is a notable lack of recent studies investigating this SNP in relation to RA, particularly in non-European populations. Our study addresses this gap by providing the first evidence of the association between CD209 promoter polymorphisms and RA susceptibility in an Egyptian cohort, highlighting the genetic contribution of these variants in a population that has been largely underexplored. Nevertheless, the study has some limitations. Being performed at a single tertiary center may limit generalizability to the broader Egyptian population. The relatively small sample size, particularly for less common genotypes, may have reduced the statistical power to detect associations. Additionally, the study focused on genotyping without functional assays, leaving the biological effects of these variants and their mechanistic link to RA to be confirmed in future *in vitro* or *in vivo* studies. Future multicentre studies with larger, diverse cohorts and functional analyses are warranted to validate and expand upon these findings.

## Conclusions

This study demonstrates a significant association between the CD209 rs4804803 (AG) polymorphism, particularly the G allele, and increased susceptibility to RA in Egyptians. The rs4804803 variant was also linked to higher disease activity and elevated laboratory markers, whereas the CD209 rs735239 polymorphism showed no correlation with RA risk. To our knowledge, this is the first study from Egypt to investigate the relationship between CD209 promoter variants and RA susceptibility. These findings elucidate genetic risk factors pertinent to the Egyptian population, establish a foundation for larger genetic screening programs, and may guide future research on gene-environment interactions and the development of tailored therapeutic strategies for autoimmune diseases.
